# University-level nutrition training in West Africa: cost and financing issues

**DOI:** 10.3402/gha.v8.29415

**Published:** 2015-11-09

**Authors:** Roger Sodjinou, William Bosu, Nadia Fanou, Noel Zagre, Félicité Tchibindat, Shawn Baker, Helene Delisle

**Affiliations:** 1 UNICEF Regional Office for West and Central Africa, Dakar, Senegal; 2 West Africa Health Organization (WAHO), Bobo-Dioulasso, Burkina Faso; 3 Department of Nutrition and Food Science, University of Abomey-Calavi, Abomey-Calavi, Benin; 4 UNICEF Cameroon Country Office, Yaoundé, Cameroon; 5 Bill and Melinda Gates Foundation, Seattle, WA, USA; 6 Department of Nutrition, Faculty of Medicine, University of Montreal, Canada

**Keywords:** nutrition, training, tuition, fees, scholarship, West Africa

## Abstract

**Background:**

There is a serious shortage of skilled nutrition professionals in West Africa. Investing in nutrition training is one of the strategies for strengthening the human resource base in nutrition. However, little is known about how nutrition training in the region is financed and the levels of tuition fees charged. The purpose of this study was to provide a comprehensive assessment about the levels of tuition fees charged for nutrition training in the West Africa region and to determine to what extent this is of reach to the average student.

**Methodology:**

The data for this study were obtained from 74 nutrition degree programs operating in nine West African countries in 2013 through semi-structured interviews during on-site visits or through self-administered questionnaires. They included the age of the programs, school ownership, tuition fees, financial assistance, and main sources of funding. Tuition fees (in 2013 US$) were expressed per program to enable uniformity and comparability. Simple descriptive and bivariate analyses were performed.

**Results:**

Results from 74 nutrition training programs in nine countries showed a wide variation in tuition fees within and between countries. The tuition fees for bachelor's, master's, and doctoral programs, respectively, ranged from 372 to 4,325 (mean: 2,353); 162 to 7,678 (mean: 2,232); and 369 to 5,600 (mean: 2,208). The tuition fees were significantly higher (*p*<0.05) in private institutions than in public institutions (mean: US$3,079 vs. US$2,029 for bachelor's programs; US$5,118 vs. US$1,820 for master's programs; and US$3,076 vs. US$1,815 for doctoral programs). The difference in the tuition fees between Francophone and Anglophone countries was not statistically significant (mean: US$2,570 vs. US$2,216 for bachelor's programs; US$2,417 vs. US$2,147 for master's programs; US$3,285 vs. US$2,055 for doctoral programs). In most countries, the tuition fees appeared to be out of reach of the average student. Recent master's programs appeared to charge higher fees than older ones. We found a significant negative correlation between tuition fees and the age of the program, after controlling for school ownership (*r*=−0.33, *p*<0.001).

**Conclusions:**

Our findings underscore the urgent need for national governments in the region to establish benchmarks and regulate nutrition training costs. In a region where the average annual gross national income (GNI) per capita is barely 890$, the rising cost of tuition fees is likely to hinder access of students from poor background to nutrition training. Governments should institute financing mechanisms such as scholarships, public–private partnerships, credit facilities, and donor funding to facilitate access to tertiary-level nutrition training in the region.

The West Africa region faces a serious shortage of skilled nutrition professionals. While it is estimated that about 2,500 nutrition graduates are needed every year in the whole region, the current output is just at 700 (1). This situation hinders many countries in the region from providing basic nutrition services or expanding the coverage of existing nutrition programs. As a result, the coverage of severe acute malnutrition treatment and other key nutrition-specific interventions (early initiation of breastfeeding, exclusive breastfeeding during the first 6 months, vitamin A supplementation of pre-school children, and iron–folic acid supplementation of pregnant women) is low in many West African countries (2). This situation also hampers progress toward achieving nutrition targets in the region (3). None of the countries in the West Africa region are on track to meet the World Health Assembly target on anemia reduction. Moreover, only two countries in the region (Liberia and Sierra Leone) are on course to meet the World Health Assembly target on stunting reduction. Likewise, only one country (Benin) is on course to meet the target on wasting reduction (3).

To address these challenges in a sustainable manner, it is imperative to bridge the current gap in manpower for the nutrition sector (4). Investing in nutrition training is one of the strategies for strengthening the human resource base in nutrition and building a critical mass of nutrition professionals in West Africa (5–7). It is, therefore, vital that governments in the region take steps to create an enabling environment that is conducive to the sustainable production of nutrition graduates. They need to facilitate access for those who want to embark on the nutrition.

This study was conducted as part of a region-wide capacity needs assessment aimed at identifying the capacities that need to be developed to accelerate progress in nutrition in the West Africa region. The assessment was done within the framework of the West African Nutrition Capacity Development Initiative (WANCDI), implemented under the auspices of the West Africa Health Organization (WAHO). The current capacities for human nutrition training in West Africa as well as the capacity to act in nutrition in the region have been described elsewhere (1, 8, 9). In this paper, we sought to examine to what extent tuition fees may hinder access to university-level nutrition training in West Africa. Although it is well established that tuition fees are one of the factors that restrict access to higher education in resource-poor settings (10), little is known about how nutrition training in the region is financed and the levels of tuition fees charged. The purpose of this study was to provide a comprehensive assessment about the levels of tuition fees charged for nutrition training in the West Africa region and determine to what extent this is of reach of the average student.

## Methods

### Data collection

Data were collected in 2013 in the West Africa region, which includes 16 countries according to the United Nations country classifications (11). These are Benin, Burkina Faso, Cape Verde, Cote d'Ivoire, Ghana, Guinea, Guinea-Bissau, Liberia, Mali, Mauritania, Niger, Nigeria, Senegal, Sierra Leone, The Gambia, and Togo.

Briefly, data were collected using an interviewer-administered or self-administered semi-structured, open-ended questionnaire (1). We established a preliminary list of institutions offering nutrition degree programs based on information provided by in-country key informants or through literature review and internet search. This list was updated until the end of the data collection period. A brief introductory email and the study questionnaire were sent to the heads of the programs to explain the objectives of the study and request their participation. Then we had a face-to-face meeting with them (or with the persons designated by them) during which we further explained the objectives of the study and administered the questionnaire. We were not able to have an in-person meeting with all respondents. When a face-to-face meeting was not possible, we asked the respondents to complete the questionnaire and return it to us by email. About 35% of the questionnaires were self-administered (1). The data collected included the age of the programs, school ownership, tuition fees for the current academic year, financial assistance, and main sources of funding.

As reported in a previous paper, 83 nutrition degree programs were operating in 10 countries of the region at the time of data collection (1). We obtained data on tuition fees for 74 nutrition degree programs operating in nine of these countries (Benin, Burkina Faso, Cote d'Ivoire, Ghana, Guinea, Niger, Nigeria, Senegal, and Sierra Leone) (Table 2). Mauritania was not included in this study as we did not get the information about tuition fees for the programs operating in the countries. The six other countries of the region that were also not included in the present study (Cape Verde, Guinea-Bissau, Liberia, Mali, The Gambia, and Togo) were those that offered no nutrition degree program at the time of data collection (1).

### Data analysis

Data were entered in MS Excel 2010, double-checked, and cleaned up. They were later exported and analyzed in SPSS 14.0. The student's *t*-test was used to determine if mean tuition fees differ according to school ownership or linguistic group of countries. The Pearson correlation test was used to assess whether there is an association between tuition fees and the age of the program, after controlling for school ownership. The level of significance was set at *p*<0.05 for all tests.

Tuition fees (including registration fees) were the only elements considered in this study. We did not collect data on other direct and indirect costs besides tuition that influences access to nutrition training. In addition, only tuition fees for national students were considered. To enable uniformity and comparability, all data on tuition fees were converted into US$ (2013 dollars). Likewise, as the duration of the programs vary from one country to another and even within the same country, entire program tuition fees were presented. However, annual tuition fees, expressed as percentage of annual gross national income (GNI) per capita, were used to assess if the costs of nutrition training were within reach of the average student in the different countries (12).

In this paper, ‘public institutions’ refers to organizations that are owned by the government and managed by a government body, regardless of the origin of their funding. In contrast, ‘private institutions’ refers to institutions that are managed by non-government affiliated organizations, regardless of their source of funding.

### Ethical considerations

All respondents were fully informed about the objectives of the assessment. They gave their full verbal consent prior to participating in the study.

## Results

### Description of the programs

The list of universities that offered nutrition degree programs in West Africa at the time of data collection is presented in Table 1. In 2013, the total student intake for these programs was estimated at 2,118 (1,610 for bachelor's programs, 471 for master's programs, and 37 for doctorate programs), while the total number of graduates in 2012 was estimated at 712 (517 for bachelor's programs, 174 for master's programs, and 21 for doctorate programs) (1). Of the total 74 programs included in this study, 17 (23%) were run by private institutions and 57 (77%) by government-supported institutions (Table 2).

**Table 1:** List of universities that provide nutrition training programs in West Africa

Country	Institution	Bachelor's programs	Master's programs	Doctorate programs
Benin	Université d'Abomey-Calavi	*Licence Professionnelle en Nutrition humaine, Sciences et Technologie Agro-alimentaires* *Licence Professionnelle en Nutrition et Diététique*	*Diplôme d'Etudes Approfondies en Nutrition et Sciences Alimentaires* *Master en Nutrition Humaine et Sécurité Alimentaire* *Master en Nutrition et Sécurité Alimentaire* *Master en Nutrition et Santé des Populations*	*Doctorat Unique en Nutrition et Sciences Alimentaires*
Burkina Faso	Université de Ouagadougou	*Licence en Nutrition et Technologie Alimentaire*	*Master en Nutrition Humaine et Toxicologie Alimentaire*	–
Cote-d'Ivoire	Université Nangui Abrogoua		*Master en Nutrition et Sécurité Alimentaire*	–
Mauritania	Université des Sciences et Technologies et de Médecine	*Licence Professionnelle en Nutrition et santé*	*Master en Nutrition Humaine, Santé et Sciences des Aliments*	–
Niger	Université Libre de Maradi	*Technicien Supérieur en Nutrition Humaine*		–
	Institut de Santé Publique	*Technicien Supérieur en Nutrition Humaine*	*Master en Nutrition Humaine*	–
	Institut Pratique de Santé Publique	*Technicien Supérieur en Nutrition Humaine*	–	–
	Ecole de Santé Publique et de l'Action Sociale	*Technicien Supérieur en Nutrition Humaine*	–	–
	Institut Privé de Santé et de Développement Communautaire	*Technicien Supérieur en Nutrition Humaine*	–	–
	Ecole de Santé Publique et de l'Action Sociale	*Technicien Supérieur en Nutrition Humaine*	–	–
	Institut L'Elite de Niamey	–	*Master en Sécurité Alimentaire et Nutritionnelle*	–
Guinea	Ecole Supérieure du Tourisme et de l'Hôtellerie	*Licence en Sciences Nutritionnelles*	–	–
Ghana	University of Ghana	BSc in Nutrition and Dietetics	MPhil Family Nutrition and Consumer Science	PhD in Human Nutrition
		BSc Family Nutrition and Consumer Science	MSc Dietetics	
		BSc Nutrition and Food Sciences	MPhil Human Nutrition	
	University of Development Studies	BSc Community Nutrition	–	–
	Kwame Nkrumah University of Sciences and Technology		MPhil Human Nutrition and Dietetics	
Nigeria	Federal University of Agriculture of Abeokuta	BSc in Nutrition and Dietetics	MSc Nutrition and Dietetics	PhD in Nutrition and Dietetics
	Kogi State University	BSc Nutrition	–	–
	Afe Babalola University	BSc Human Nutrition and Dietetics	–	–
	University of Ibadan	BSc Human Nutrition and Dietetics	MPhil Human Nutrition M.Sc. Human Nutrition	PhD Human Nutrition
	Obafèmi Awolowo University	BSc Family Nutrition and Consumer Sciences	–	–
	Babcock University	BSc (Hons) Nutrition and Dietetics	MPhil Community and Human Nutrition MSc Nutritional Biochemistry	PhD Nutritional Biochemistry PhD Community and Human Nutrition PhD Medical Dietetics
	University of Nigeria Nsukka	BSc Nutrition and Dietetics	MSc Human Nutrition	PhD Human Nutrition
			MSc Nutrition and Food Biochemistry	
	Novena University	BSc Nutrition and Health studies	–	–
	Wesley University of Science and Technology	BSc Human Nutrition and Dietetics	–	–
	Fountain University	BSc (Hons) Biochemistry and nutrition	–	–
	Michael Okpara University of Agriculture	BSc Nutrition and dietetics	MSc Nutrition and dietetics (Applied Human Nutrition, community Nutrition and experimental Nutrition)	PhD Applied Human Nutrition, Community
			MSc Nutritional Biochemistry	
	Imo State University	BSc Nutrition and Dietetics	–	–
	Ahmadu Bello University	BSc Nutritional Biochemistry	MSc Human Nutrition	PhD Human Nutrition
			MSc Nutritional Biochemistry	
	University of Mkar	BSc Home Science, Nutrition and Dietetics	–	–
	Nmamdi-Azikiwe University Teaching Hospital	BSc Nutrition and Dietetics	–	–
	University of Calabar		MSc Nutrition and Food Science	PhD Nutrition and Food Science
	University of Port Harcourt	–	MSc Nutrition and Toxicology	PhD Nutrition and Toxicology
			Master Nutrition Physiology Engineering	
	Ambrose Alli University	–	MSc Nutritional Biochemistry	PhD Nutritional Biochemistry
	University of Jos	–	MSc Nutritional Biochemistry	–
	Madonna University	–	MSc Nutrition and Dietetics	PhD Nutrition and Dietetics
	Abia State University	–	MSc Nutritional Biochemistry	PhD Nutritional Biochemistry
	Alvan Ikoku Federal College of Education	–	–	PhD Applied Nutrition
Senegal	Université de Alioune Diop	–	*Master en Nutrition Communautaire*	
	Université Cheikh Anta Diop	–	*Master en Nutrition et Alimentation Humaine*	*Doctorat en Nutrition et Alimentation Humaine*
Sierra Leone	Njala University of Sierra Leone	BSc (Hons) Nutrition and Dietetics	MSc Nutrition and Dietetics	–
		BSc (Hons) Nutrition and Food Technology	–	–

**Table 2:** Number of training programs included in the analysis

	Number of programs according to the level of degree				Number of programs according to the school ownership	
		
Country	Bachelor	Masters	Doctorate	Total	Programs run by private institutions	Programs run by public institutions
Benin	2	4	1	7	0	7
Burkina Faso	1	1	0	2	0	2
Cote-d'Ivoire	0	1	0	1	0	1
Ghana	4	4	1	9	0	9
Guinea	1	0	0	1	0	1
Niger	5	2	0	7	5	2
Nigeria	11	17	13	41	12	29
Senegal	0	2	1	3	0	3
Sierra Leone	2	1	0	3	0	3
Total	26	32	16	74	17	57

### Undergraduate nutrition degree programs

The mean tuition fees for bachelor's programs were US$2,353 (range: 372–4,325) (Table 3). Most of the programs (57.7%, 15/26) charged over US$2,000, 27% (7/26) charged between US$1,000 and US$2,000, and the remaining 15.4% (4/26) charged below US$1,000. Tuition fees were significantly higher in private institutions than in public institutions (mean: US$3,079 vs. US$2,029, *p*<0.05) for bachelor's programs (Table 4). The difference in the entire program tuition fees charged between Francophone and Anglophone countries (mean: US$2,570 vs. US$2,216) was not statistically significant (Table 4).

**Table 3:** Mean tuition fees and average annual GNI per capita in different countries (US$)

Country	Bachelor	Master	Doctorate	Average annual GNI per capita for the country^a^
Benin	3,415 (2,505–4,325)	2,824 (2,035–6,091)	5,553	1,147
Burkina Faso	1,523^b^	1,015^b^	–	1,326
Cote-d'Ivoire	–	244^b^	–	1,475
Ghana	2,085 (1,095–2,804)	5,524 (5,489–5,600)	5,600^b^	2,601
Guinea	1,748^b^	–	–	1,986
Niger	2,528 (2,437–2,741)	3,472 (2,030–4,914)	–	896
Nigeria	1,339^c^ (372–4,309)	558 (202–7,678)	1,024 (369–5,340)	1,188
Senegal	–	1,096 (162–2,030)	1,036	1,914
Sierra Leone	4,309^b^	401^b^	–	901
Total (mean, range)	2,353 (372–4,325)	2,232 (162–7,678)	2,208 (369–5,600)	–

–: No training program was offered at this level; (): range; GNI, gross national income.

a Data were derived from Ref. (12). GNI Purchasing-Power Parity was used;

b only one nutrition degree program was offered;

c there were no tuition fees for undergraduate students in public universities in Nigeria.

**Table 4:** Mean tuition fees (US$) by school ownership and linguistic group of countries

Category	Response	Bachelor	*p*	Masters	*p*	Doctorate	*p*
Ownership	Private	3,079	<0.05	5,118	<0.05	3,076	<0.05
	Public	2,029		1,820		1,815	
Linguistic group^a^	Francophone	2,570	NS	2,417	NS	3,285	NS
	Anglophone	2,216		2,147		2,055	

NS=not statistically significant.

a Francophone countries: Benin, Burkina Faso, Cote d'Ivoire, Guinea, Niger, and Senegal. Anglophone countries: Ghana, Nigeria, and Sierra Leone.

The tuition fees varied widely between programs within the same country and between countries (Table 3). The mean tuition fees for bachelor's programs varied from US$1,523 in Burkina Faso to US$4,309 in Sierra Leone. The within-country variation was widest in Nigeria (US$372–US$4,309) and narrowest in Niger (US$2,437–US$2,741).

**Fig. 1:**
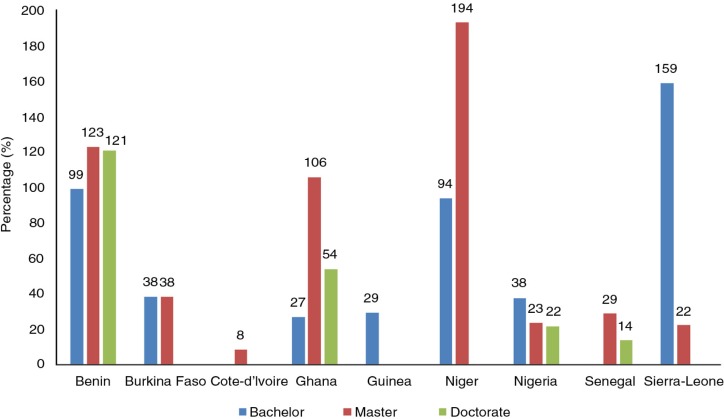
Annual tuition fees charged in the different programs as a percentage of annual gross national income (GNI) per capita. Data on GNI were derived from Ref. (12).

The annual tuition fees charged in the bachelor's programs ranged from 27 to 159% of annual GNI per capita (Fig. 1). The cost burden of tuition fees on income was highest in Sierra Leone (159% of GNI), Benin (99%), and Niger (94%), and lowest in Ghana (27% of GNI) and Guinea (29%).

### Master's programs in nutrition

The mean tuition fees for master's programs were US$2,232 (range: 162–7,678) (Table 3). About 46.9% (15/32) of the programs charged below $1,000, 40.6% (13/32) over US$2,000 and the remaining 12.5% (4/32) between US$1,000 and US$2,000. As with the bachelor's programs, the mean tuition fees were significantly higher in private institutions than in public institutions (mean: US$5,118 vs. US$1,820, *p*<0.05) for master's programs (Table 3). There was also no significant difference in the mean tuition fees charged between Francophone and Anglophone countries (mean: US$2,417 vs. US$2,147).

As with the bachelor's programs, there was also a wide variation in the tuition fees for master's programs within the same country and between countries (Table 3). The mean tuition fees for master's programs varied from US$401 in Sierra Leone to US$5,524 in Ghana. The within-country variation in tuition fees was widest in Nigeria (US$202–US$7,678) and Senegal (US$162–US$2,030), and narrowest in Ghana (US$5,489–US$5,600).

The annual tuition fees charged in the master's programs ranged from 8 to 194% of annual GNI per capita (Fig. 1). These fees appeared to be out of reach for most people in Niger (194% of GNI), Benin (123%), and Ghana (106%). Lower figures were, however, found in Cote-d'Ivoire (8% of GNI), Sierra Leone (22%), Nigeria (23%), Senegal (29%), and Burkina Faso (38%).

We found a significant negative correlation between actual tuition fees for master's programs and the age of the program, after controlling for ownership of institution (*r*=−0.33, *p*<0.001). The more recent the master's program, the higher the tuition fees charged. However, we found no correlation in the undergraduate and doctoral programs.

### Doctorate degree programs in nutrition

The mean tuition fees for doctoral programs were US$2,208 (range: 369–5,600) (Table 3). Overall, 37.5% (6/16) of the programs charged below $1,000, 31.3% (5/16) charged over US$2,000, and the remaining 31.3% (5/16) charged between US$1,000 and US$2,000 per year. As with the bachelor's and master's programs, the mean tuition fees for doctoral programs were significantly higher in private institutions than in public institutions (mean: US$3,076 vs. US$1,815, *p*<0.05) (Table 3). There was no significant difference between Francophone and Anglophone countries (mean: US$3,285 vs. US$2,055) with respect to tuition fees charged.

The mean tuition fees for doctoral programs varied from US$1,024 in Nigeria to US$5,600 in Ghana (Table 3). There was also a wide variation in tuition fees within countries, with the widest variation being observed in Nigeria (US$369–US$5,340).

The annual tuition fees charged in the doctoral programs ranged from 14 to 121% of annual GNI per capita (Fig. 1). The cost burden of tuition fees on income was highest in Benin (121% of GNI) and lowest in Senegal (14% of GNI).

The mean tuition fees were about the same or decreased slightly with increasing level of training (Table 3). However, within countries, the fees increased with increasing level of training in Ghana and Niger. A U-shaped pattern was observed in Benin and Nigeria.

## Discussion

In this study, we provide a comprehensive assessment about the levels of tuition fees charged for nutrition training in the West Africa region. Our results indicate a disparity in tuition fees charged for nutrition degree programs within and between countries. Currently, there is little regulation of the tuition fees charged for nutrition degree programs in the region, especially those run by private institutions. The government of Nigeria makes it mandatory for public universities to adopt a policy of no tuition fees for undergraduate students, but it has no control over the tuition fees charged by private institutions (13). As nutrition training programs are being developed, there is an urgent need for governments of each country in the region to establish benchmarks and regulate nutrition training costs, from the perspective of promoting equity and diversity in access to university-level nutrition training. This will facilitate not only access to nutrition training but also the mobility of students across the region.

We found that private institutions offering nutrition programs charge higher fees than public ones. The fact that private institutions charge higher fees than public institutions is understandable as they had very little government support and relied heavily on tuition fees (1). Whereas it is essential for governments in the region to pursue (or maintain) their support to programs run by public institutions (1), part of their investments should also be directed to private training institutions because of the public good nature of nutrition. Governments are the end-users of the nutrition graduates, produced by both public and private institutions, who will help to move their national nutrition agenda forward. Additional support should be sought from bilateral donors and non-governmental organizations.

We found a rising trend in tuition fees for master's degree programs. Newer programs appeared to charge higher fees than older ones, probably because of a global trend toward higher fees. The increasing price of tuition fees for nutrition training is an issue of great concern. Interestingly, higher tuition fees do not necessarily equate to quality training (14). It is critical to maintain an appropriate balance between quality and affordability. Upgrading the quality of nutrition training in the West Africa region is indeed needed. Training institutions in the region are facing critical human and financial challenges (critical shortage of faculty members, lack of public funding, lack of equipment and infrastructure, etc.) which hamper their abilities to provide quality nutrition training (1). As pointed out by the World Bank (15), poor-quality training in the sector of higher education in sub-Saharan Africa is leading to the production of graduates who are poorly equipped to compete on the job market or tackle the challenges that their countries are facing. The quest of quality, however, should not be made at the expense of affordability. Whereas it is essential to ensure high-quality standards, it is also important for nutrition training in West Africa to be affordable to prospective nutritionists. If tuition fees continue to increase, it is likely that many students, especially those from deprived backgrounds, will be denied access to nutrition education. This has previously been reported for dental and medical education (16, 17). This situation can also result in inequalities in the social system as seen in Nigeria where students from poor families cannot afford to embark on the programs offered by private universities (18). Governments should therefore take steps to provide a comprehensive framework for nutrition training that guarantee quality standards and at the same time affordability. As the countries in the region differ greatly in terms of economic situation, solutions need to be adapted to specific contexts. There is therefore no ‘one size fit all’ solution for this issue. Various financing options need to be explored. This could include public financing, public–private partnerships, scholarship schemes, loan programs, and generation of additional resources by the universities (15). In a region where the average annual GNI per capita is barely 890$ (12), public financing of nutrition training is one of the possible solutions (18), even if public funding in the region is already overstretched (15). Governments in the region should explore opportunities for private–public partnerships and donor funding. They should also offer scholarship opportunities to talented students. Moreover, governments need to take steps to establish loan programs and make credit facilities for students. Currently, they are only two countries in the region (Nigeria and Ghana) that have full-fledged loan programs for tertiary education (15). Whereas it is essential for governments to increase their investments and support for nutrition training, the universities themselves should raise money and mobilize additional resources through approaches such as the development of in-service and continuing training courses in nutrition, external collaborations, and consultancy services (15). For example, all federal universities in Nigeria are required to mobilize part of their annual budget internally through various means (19). Additionally, the bachelor's programs should charge lower fees than graduate programs considering the type of training needed and the fact that undergraduates should form the base of the nutrition workforce pyramid (6, 20). Presently, more master's level nutritionists are produced than undergraduate-level nutritionists in French-speaking West Africa (1).

Our study has some limitations. While great effort was made to assure total coverage, we could not cover all the training programs that may exist in the region. The data for the programs analyzed may therefore not be representative of all the programs in some countries. In addition, the focus was only on nutrition degree programs in this study. In-service nutrition training as well as non-university technical training, although equally important, was beyond the scope of this paper. Moreover, we could only obtain the data on tuition fees for 74 of the 83 nutrition degree programs that were found at the time of the survey. Additionally, we did not collect data on other direct and indirect costs besides tuition fees that influence access to nutrition training. Further research on this may be warranted. Finally, we used GNI per capita as a proxy for average income in the different countries. This may not reflect the true situation as indicated by the World Bank (15). These limitations notwithstanding, our findings reflect to a large extent the range and trend in tuition costs for nutrition training in the region.

## Conclusions

The tuition fees charged by training institutions offering nutrition degree programs in West Africa appeared to be out of reach for the average student in most countries. These fees were significantly higher in private institutions than in public ones. This calls for wider policy reforms on the financing of nutrition training in the region. Governments in the region need to take steps to establish or enforce benchmarks and regulate nutrition training costs, particularly in private institutions. The rising cost of tuition fees in West Africa is likely to limit access of students from a poor background to nutrition training. Governments should also explore innovative financing mechanisms that facilitate access to nutrition training and guarantee at the same time quality standards. It is critical to establish and institutionalize loan programs for tertiary-level nutrition training in the region. Foreign assistance (bilateral or multilateral) should also contribute to strengthening of local programs through providing scholarships for in-country training.
